# De novo transcriptome sequencing and analysis of male and female swimming crab (*Portunus trituberculatus*) reproductive systems during mating embrace (stage II)

**DOI:** 10.1186/s12863-017-0592-5

**Published:** 2018-01-03

**Authors:** Zhengfei Wang, Linxia Sun, Weibing Guan, Chunlin Zhou, Boping Tang, Yongxu Cheng, Jintian Huang, Fujun Xuan

**Affiliations:** 1 Jiangsu Key Laboratory for Bioresources of Saline Soils, Jiangsu Synthetic Innovation Center for Coastal Bio-agriculture, Jiangsu Provincial Key Laboratory of Coastal Wetland Bioresources and Environmental Protection, School of Ocean and Biological Engineering, Yancheng Teachers University, Yancheng, 224001 Jiangsu Province People’s Republic of China; 2 Key Laboratory of Shanghai Education Commission for Oceanic Fisheries Resources Exploitation, Shanghai Ocean University, Shanghai, 200090 People’s Republic of China; 3 Key Laboratory of Freshwater Aquatic Genetic Resources, Ministry of Agriculture, Shanghai Ocean University, Shanghai, 200090 People’s Republic of China; 4 Yancheng Institute of Technology, Yancheng, 224051 People’s Republic of China

**Keywords:** Reproductive systems, Differentially expressed genes, Transcriptome, *Portunus Trituberculatus*

## Abstract

**Background:**

The swimming crab *Portunus trituberculatus* is one of the most commonly farmed crustaceans in China. As one of the most widely known and high-value edible crabs, it crab supports large crab fishery and aquaculture in China. Only large and sexually mature crabs can provide the greatest economic benefits, suggesting the considerable effect of reproductive system development on fishery. Studies are rarely conducted on the molecular regulatory mechanism underlying the development of the reproductive system during the mating embrace stage in this species. In this study, we used high-throughput sequencing to sequence all transcriptomes of the *P. trituberculatus* reproductive system.

**Results:**

Transcriptome sequencing of the reproductive system produced 81,688,878 raw reads (38,801,152 and 42,887,726 reads from female and male crabs, respectively). Low-quality (quality <20) reads were trimmed and removed, leaving only high-quality reads (37,020,664 and 41,021,030 from female and male crabs, respectively). A total of 126,188 (female) and 164,616 (male) transcripts were then generated by de novo transcriptome assembly using Trinity. Functional annotation of the obtained unigenes revealed that a large number of key genes and some important pathways may participate in cell proliferation and signal transduction. On the basis of our transcriptome analyses and as confirmed by quantitative real-time PCR, a number of genes potentially involved in the regulation of gonadal development and reproduction of *P. trituberculatus* were identified: *ADRA1B*, *BAP1*, *ARL3*, and *TRPA1*.

**Conclusion:**

This study is the first to report on the whole reproductive system transcriptome information in stage II of *P. trituberculatus* gonadal development and provides rich resources for further studies to elucidate the molecular basis of the development of reproductive systems and reproduction in crabs. The current study can be used to further investigate functional genomics in this species.

**Electronic supplementary material:**

The online version of this article (10.1186/s12863-017-0592-5) contains supplementary material, which is available to authorized users.

## Background

The swimming crab (*Portunus trituberculatus*) is a commercially important crab species for both aquaculture and fisheries widely distributed in East Asian countries [1]. In China, this species spreads widely throughout marginal seas, including the East China Sea, South China Sea, Yellow Sea, and Bohai Sea [2]. *P*. *trituberculatus* is one of the most important marine species cultured in China because of its high nutritional content and economic value. This crab has a life cycle of 2–3 years and can reach sexual maturity in the first year [3, 4]. Before the puberty molt, *P*. *trituberculatus* grows rapidly by frequent molting (every 5–30 d) [3]. Immediately after puberty molt and mating, the female *P. trituberculatus* usually starts vitellogenesis and ovarian development [3, 5]. During these stages, the development of the reproductive system is vital to *P. trituberculatus* because not only are many larval crabs needed for aquaculture, an individual with a mature ovary is more widely known to consumers as well.

The reproductive system is fundamental to reproductive processes [6]. The reproductive system of the male crab contains paired testes, vas deferens, seminal vesicles, and a genital aperture, whereas the female crab consists of paired ovaries, oviducts, gonophores, and an external sperm reception area [7]. The development of the male and female reproductive system is crucial to the reproduction as well as commercial seed production of *P. trituberculatus*. The gonads (testes and ovaries) are the primary reproductive organs, and their development affects reproduction and sex differentiation [8]. Gonadal development consists of ovarian and testicular development and maturation, such as spermatogenesis, ovarian differentiation, and vitellogenesis. In male *P. trituberculatus*, spermatogenesis is mostly characterized by the differentiation of sperm cells and their maintenance before fertilization. In female *P. trituberculatus*, ovarian development is usually divided into 6 stages [5]. In stage I, the ovary is transparent and ribbonlike, and the main cells consist of oogonia and previtellogenic oocyte. In stage II (mating embrace), the ovary is milky white, and the main cells are composed of endogenous vitelloge and previtellogenic oocytes. In stage III, the ovary is buff and orange, and the main cell type is an exogenous vitellogenic oocyte. In stage IV, the main cell types are exogenous vitellogenic and nearlymature oocytes in the ovary, which turn into deep orange. In stage V, the ovary is ripe, and the main cell type is a mature oocyte. Finally, in stage VI, the crab has spawned, and the ovary turns light orange [5, 9, 10].

The ovarian development of *P.trituberculatus* is initiated by copulation. When female pubertal molting and subsequent mating occur (stage II), the ovary remains milky white, and the main cell types are endogenous vitellogenic and previtellogenic oocytes [5, 9, 10]. Meanwhile, the special structure of the sperm plug is also formatted in the spermatheca during mating. In a mating embrace (stage II), seminal fluid proteins (including protease inhibitors, lectins, prohormones, peptides, and protective proteins such as antioxidants) are transferred to females with sperm [9, 11]. Thus, to understand the regulatory mechanisms underlying reproductive development in this species during stage II, both whole reproductive systems should be considered together. The reason is that an increasing number studies show that male seminal material performs vital functions in female reproductive fitness, including ovarian development and sperm plug formation [12, 13]. However, no studies identifying and characterizing stage II in *P. trituberculatus* have been conducted at the transcriptome level.

Transcriptome is a collection of transcripts within cells in certain physiological conditions [14], and transcripts can be used to examine the expression of genes at the RNA level. Transcriptome studies have rapidly emerged in recent years following the advent of next-generation sequencing technology, which provides high-throughput and low-cost sequencing that exhibits significant influence on genetic research. A number of studies have analyzed the gonad transcriptome and provided information about the molecular mechanisms underlying gonadal development and maturation [15–17]; but few have focused on the reproductive system. Various sequencing platforms have also been established, such as Solexa illumine, Roche 454, and ABI solid [18–20]. These technologies have been applied in studying the transcriptome of many aquatic animals, particularly in crustaceans [18–20].

In the present study, de novo assembly and high-throughput Illumina HiSeq sequencing were employed to obtain the comparative transcriptome of the female and male reproductive systems of *P. trituberculatus* during the mating embrace (stage II). Moreover, differentially expressed genes (DEGs) were identified and analyzed. The current study intends to quantify and identify spermatogenesis and ovarian differentiation related genes and identify the pathways involved. Findings from this transcriptome dataset can provide valuable resources to elucidate the molecular mechanisms underlying the physiological and morphological changes during stage II of the development of the whole reproductive system. Thus, the current study can provide valuable genomic information to understand the development of reproductive systems and the maturation of *P. trituberculatus*.

## Results

### Illumina sequencing and assembly

To obtain an overview of the reproductive system transcriptome of *P. trituberculatus* and identify the genes involved in the development and maturation of the reproductive systems, two cDNA libraries were prepared from pooled RNA extracts of female and male reproductive systems in stage II. These extracts were then sequenced using the Illumina Solexa platform. A total of 42,887,726 and 38,801,152 raw reads were generated from male and female transcriptome sequencing, respectively. Ambiguous nucleotides and low-quality (quality <20) reads were trimmed and removed. The remaining high-quality reads, totaling to 41,021,030 and 37,020,664 reads from male and female, respectively, were then obtained. All reads were submitted to the website of the National Center for Biotechnology Information Short Read Archive site, with accession number SRR5723730 and SRR5723731.

**Fig. 1:**
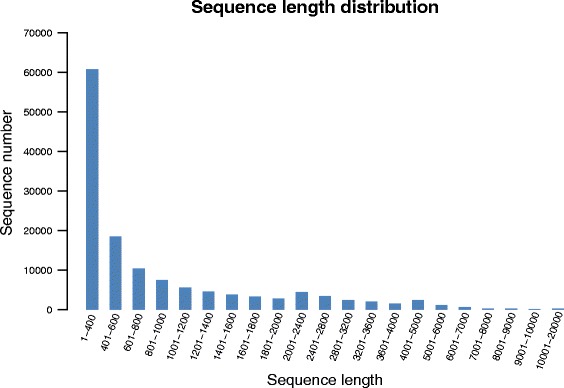
Length distribution of the final assembled unigenes. The X axis shows the sequence lengths of the unigenes, and the Y axis shows the number of unigenes

De novo transcriptome assembly with Trinity using reads from female and male crabs generated 126,188 and 164,616 transcripts, respectively. The average length of the transcripts in the female and male crabs were 954 bp (ranging from 201 bp to 20,074 bp) and 823 bp (ranging from 201 bp to 17,641 bp), respectively. The detailed sequencing and assembly results for the female and male crabs are shown in Table 1. We used Illumina sequencing to generate a substantial number of long sequences; the lengths of 17.25% (23, 457) of the total transcripts exceeded 600 bp, and the lengths of 4.13% (5, 613) of the transcripts exceeded 1, 000 bp (Fig. 1).

**Table 1:** Result of the de novo transcriptome assembly performed with Trinity

	Type	Unigene	Transcripts
Female	Total sequence num	70,807	126,188
	Total sequence base	50,955,454	120,326,367
	Percent GC	45.04	45.79
	Largest	20,074	20,074
	Smallest	201	201
	Average	719.64	953.55
	N50	1268	1839
Male	Total sequence num	101,401	164,616
	Total sequence base	63,867,553	135,502,734
	Percent GC	46.30	46.58
	Largest	17,641	17,641
	Smallest	201	201
	Average	629.85	823.14
	N50	934	1504

### Annotation of unique sequences

**Fig. 2:**
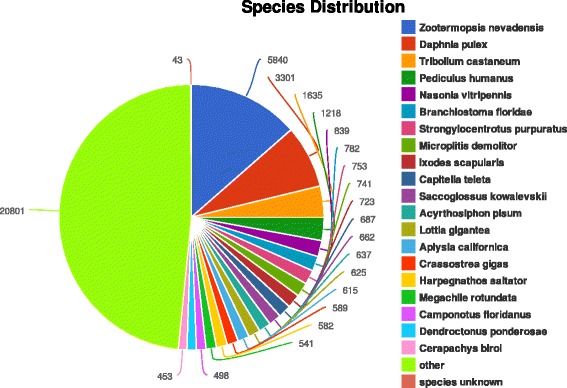
Species distribution of BLASTx matches the reproductive system transcriptome unigenes. Each piece of fan indicates the number of top BLAST matches (the matches with the lowest E-value for each unigene) against the Genbank non-redundant (Nr) protein database for various species

Annotation of 88,804 unigenes was conducted using BLASTX [21] searches against the String, KEGG, Pfam, Swissprot, and non-redundant (NR) databases. The numbers of matched unigenes in the respective databases were as follows: 4021 (4.53%), String database; 8432 unigenes (9.50%), KEGG; 10,924 unigenes (12.30%), Pfam; 11,887 unigenes (13.39%), Swissprot, and 17,558 unigenes (19.77%), NR. The BLASTX top-hit species distribution of the 1928 annotated unigenes exhibited the highest homology to *Zootermopsis nevadensis* (11.34%), followed by *Daphnia pulex* (1137, 6.69%), *Tribolium castaneum* (657, 3.87%), and *Pediculus humanus* (439, 2.58%) (Fig. 2).

### Functional annotation of the transcriptome

**Fig. 3:**
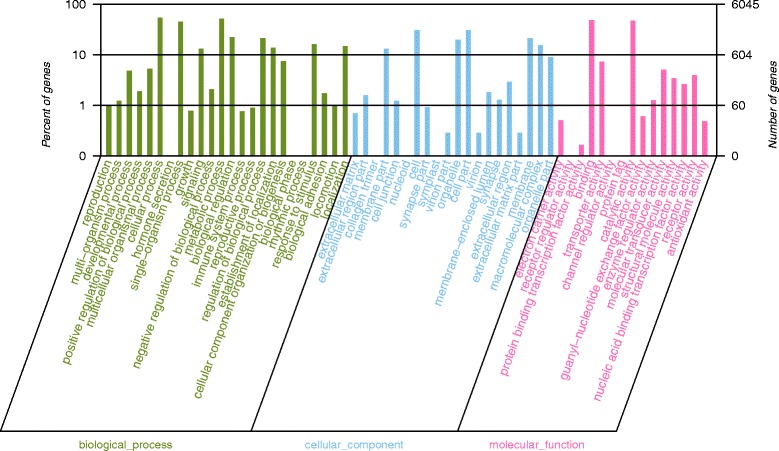
Gene ontology (GO) assignment of assembled unigenes of *P. trituberculatus*. GO terms were processed by Blast2Go and categorized at the 2nd level under 3 main categories (biological process, cellular component, and molecular function)

To functionally categorize the assembled unigenes, gene ontology (GO) assignment programs were used. The functions of 6046 annotated sequences were predicted. Blast2 GO was used to assign the GO terms. These unigenes were assigned to major GO categories (level 3), i.e., biological process, cellular component, and molecular function (Fig. 3). For biological processes, “cellular process” (54.00%, GO: 0009987) was the most abundant term. The genes involved in the metabolic process (51.10%, GO: 0008152), single-organism process (44.81%, GO: 0044699), organic substance metabolic process (39.52%, GO: 0071704), primary metabolic process (38.54%, GO: 0044238), single-organism cellular process (37.24%, GO: 0044763), and cellular metabolic process (36.20%, GO: 0044237) were also highly represented. For the cellular component, “cell” (30.67%, GO: 0005623) and “cell part” (30.67%, GO: 0044464) were the most represented categories, followed by “intracellular” (26.62%, GO: 0005622), “intracellular part” (24.47%, GO: 0044424), “membrane” (21.09%, GO: 0016020), and “organelle” (19.90%, GO: 0043226). For molecular function, “binding” (47.58%, GO: 0005488) was the most prevalent, followed by “catalytic activity” (47.06%, GO: 0003824), “organic cyclic compound binding” (28.59%, GO: 0097159), “heterocyclic compound binding” (28.55%, GO: 1,901,363), and “ion binding” (25.69%, GO: 0043167) (Fig. 3). Among all GO terms, the biological process ontology comprised the largest proportion, followed by the molecular functions ontology and the cellular component ontology. Within the biological process ontology, 288 unigenes were identified as developmental process (GO: 0032502), 57 unigenes were annotated to reproduction (GO: 0000003), and 47 unigenes were annotated to growth (GO: 0040007).

**Fig. 4:**
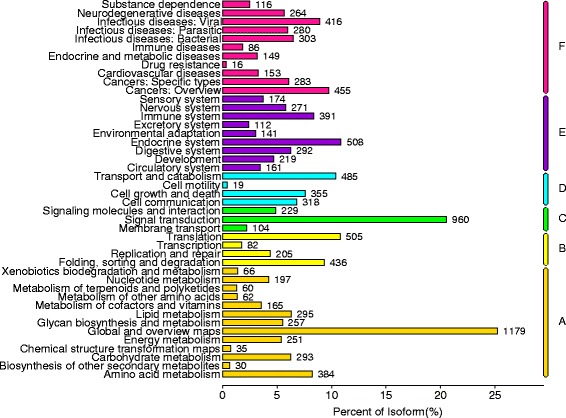
Distribution of the mapped Kyoto Encyclopedia of Genes and Genomes (KEGG) pathways

To further analyze the possible pathways involved in the development of the *P. trituberculatus* reproductive system, all unigenes were mapped to the reference pathways in the KEGG database. A total of 8, 432 unigenes were mapped to 337 pathways representing biological systems involved in stage II of the development of the *P. trituberculatus* reproductive system. The most abundant pathways represented in the reproductive systems are the metabolic pathway (1, 112 unigenes, ko01100) and the biosynthesis of secondary metabolites (299 unigenes, ko01110) (Additional file 1: Table S1 and Fig. 4). Many unigenes were mapped to pathways related to cell proliferation and signal transduction, including purine metabolism (185 unigenes, ko00230), the cAMP signaling pathway (171 unigenes, ko04024), the PI3K-Akt signaling pathway (163 unigenes, ko04151), regulation of the actin cytoskeleton (160 unigenes, ko04810), the Rap1 signaling pathway (152 unigenes, ko04015), RNA transport (151 unigenes, ko03013), protein processing in the endoplasmic reticulum (150 unigenes, ko04141), spliceosome (144 unigenes, ko03040), ribosome (133 unigenes, ko03010), lysosome (132 unigenes, ko04142), and ubiquitin-mediated proteolysis (131 unigenes, ko04120) (Additional file 1: Table S1 and Fig. 4).

### Analysis of differentially expressed genes between female and male reproductive systems

To identify and characterize the DEGs between the female and male in stage II, we found 9, 066 genes to be significantly differentially expressed (FDR < =0.01, |logFC| > =1) (Additional file 2: Table S2). Among these DEGs, 2, 723 were upregulated in the female and 6, 343 were downregulated (Additional file 3: Table S3). GO and KEGG analyses were also performed to understand the functions of these DEGs. These DEGs were then assigned to 37 GO terms and 184 pathways through the KEGG database. DEGs were mostly enriched in molecular function and biological process, and many DEGs were related to gametogenesis and signal transduction, such as genes associated with transport, transporter activity, signal transducer activity, ion channel activity, and so on. Of the functional pathways, neuroactive ligand–receptor interaction and purine metabolism were most specifically expressed in *P. trituberculatus*. In addition, for specifically expressed genes in reproductive systems, several pathways were found associated with gonadal development and sex maintenance, such as homologous recombination, the Wnt signaling pathway, and progesterone-mediated oocyte maturation.

**Fig. 5:**
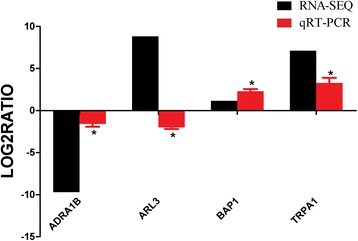
qRT-PCR validation of differentially expressed genes analyzed by RNA-seq in female. qRT-PCR was performed for 4 genes that were identified as differentially expressed between the female and male reproductive systems. The Y axis shows the relative mRNA expression levels. * *p* < 0.05

To validate the expression profiles obtained from Illumina sequencing analysis, 4 DEGs that play key roles in regulating gonadal differentiation and development were chosen for qRT-PCR analysis, using the same RNA samples. These DEGs include alpha-1B adrenergic receptor (*ADRA1B*), BRCA1-associated protein 1 (*BAP1*), ADP-ribosylation factor-like 3 (*ARL3*), and transient receptor potential ankyrin 1 (*TRPA1*). Among these, the differential expression patterns of 3 genes are consistent with the results obtained by Illumina sequencing (Fig. 5). The qRT-PCR results generally agreed with the deep sequencing analysis, which verified the data obtained by Illunima sequencing.

In addition, to study the regulatory mechanisms underlying the reproductive development of *P. trituberculatus* during the mating embrace (stage II), we set the immature crabs as the control group for expression analysis. Increased expression levels in the 4 genes (*ADRA1B*, *BAP1*, *ARL3*, and *TRPA1*) were detected in the male mature crabs, relative to those of immature crabs (Additional file 4: Figure S1). In the female mature crabs, the expression of the *ARL3* gene was significantly decreased, whereas the expression levels of the *ADRA1B*, *BAP1*, and *TRPA1* genes increased (Additional file 5: Figure S2).

## Discussion

Reproductive systems are important in the development of species and fulfill many pivotal functions, including gametogenesis, fertilization, and hormone secretion [7]. Numerous studies focused on gonad development and sexual differentially expressed genes and marker information; however, those study subjects were limited to gonad tissues (i.e., testis and ovary) [15–17]. In the present study, we performed de novo assembly of the transcriptome of the whole reproductive systems of female and male *P. trituberculatus*. Compared with those of separate gonad tissues, the transcriptomes of whole reproductive systems can offer more comprehensive information for research in gonadal development and reproduction.

The results of large-scale transcriptome sequencing of the swimming crab (the whole male and female reproductive systems) could provide resources for gene expression profiling studies as well as identification of functional classifications, molecular markers, and molecular pathways. In general, the high quality of long sequences enables us to gain more information about genes [15]. In the current study, 23,457 transcripts (17.25%) had sequence lengths exceeding 600 bp, and 5613 transcripts (4.13%) had sequence lengths exceeding 1000 bp. Therefore these datasets can provide a valuable resource for future studies on specific processes, functions, and pathways in the mating embrace (stage II) of *P. trituberculatus*.

The reproductive tissue development of crustaceans is deemed to be a dynamic process involving coordinated interactions between regulators that assemble or edit the cellular constituents supporting the developing gametes [17]. GO and KEGG analyses were used for gene function classification and annotation, and for obtaining background knowledge of gene functions that can help predict the role of protein interaction networks in cells [22]. We obtained GO and KEGG assignment results partially similar to the previous crab transcriptome sequencing; however, a large number of key genes and several important pathways may participate in cell proliferation and signal transduction (Figs. 3 and 4). Gametogenesis is a complex process involving cellular transformations that result in the production of male and female haploid germ cells, and it starts from the mitotic cell cycle followed by entry into meiosis and the completion of complex differentiation programs [23]. Signal transduction is the process by which a chemical or physical signal is transmitted through a cell as a series of molecular events, and this process is also critical for the timing and maintenance of normal gametogenesis [24]. In many arthropods, mating initiates a behavioral and physiological switch in females, triggering responses in several processes related to fertility [9]. During a mating embrace (stage II), seminal fluid proteins are transferred to the female species, inducing numerous physiological and behavioral post-mating changes in females [9]. Thus, gametogenesis and signal transduction could potentially be a primary precondition in stage II of *P. trituberculatus*.

*ADRA1B*, which targets the neuroactive-ligand receptor interaction pathway, is a member of the G protein-coupled receptor superfamily. This receptor activates phosphatidylinositol hydrolysis, which may be crucial in mitogenesis and regulates growth and proliferation in many cells [25]. Previous studies have shown that *ADRA1B* controls male fertility, spermatogenesis, and the steroidogenic capacity of Leydig cells [26]. *ADRA1B* in adult golden hamsters affects the responsiveness of testicular steroidogenesis to catecholamines [27, 28]. These findings are consistent with our current findings. In our transcriptome analysis and qRT-PCR, *ADRA1B* showed increased expression during stage II and higher transcripts in the male species than in the female species (Figs. 5 and Additional file 4: Figure S1), suggesting that this gene participates in the spermatogenesis and male fertilization of *P. trituberculatus*.

ADP-ribosylation factor-like 3 (*ARL3*), a member of the ADP-ribosylation factor family of GTP-binding proteins, can bind guanine nucleotides but lacks ADP-ribosylation factor activity [29]. In previous studies, the expression and function of *ARL3* during spermiogenesis were examined, and its potential importance in the regulation of male fertility and infertility was determined [30]. In mouse, *ARL3* was found to be expressed from birth, and the expression increased significantly when spermiogenesis began [31]. Thus, *ARL3* as a novel manchette-related protein with an important role in the spermiogenesisand formation of sperm tail collar, was usually identified as one of the potential proteins involved in the initiation of spermatogenesis [30, 31]. In our qRT-PCR, *ARL3* was significantly increased and highly expressed in the male crabs during stage II (Figs. 5 and Additional file 4: Figure S1), suggesting that this protein can participate in the spermatogenesis of *P. trituberculatus*. Notably, a decrease in the expression of the *ARL3* gene was detected in female mature crabs relative to the immature crabs (Additional file 5: Figure S2), suggesting that the *ARL3* gene played an important role in the reproductive system of male *P. trituberculatus*.

*TRPA1* is a membrane-associated cation channelthat is widely expressed in neuronal cells and involved in nociception and neurogenic inflammation [32]. Normally, *TRPA1* is activated and can cause an influx of cation ions, particularly Ca^2+^, into the activated cells; this increase in intracellular Ca^2+^ would trigger an action potential in neuronal cells [33]. *TRPA1* was unexpectedly expressed in some non-neuronal cells, such as keratinocytes, synoviocytes, and gonads [34, 35]. To illustrate, *TRPA1* is conserved between many invertebrates, except as a chemosensor for noxious compounds and as a sensor for temperature-driven behaviors [33, 36–38]. Given that high temperature can simulate growth and initiate early ovarian development [39], *TRPA1* may be activated by temperature fluctuations in the ovary. In the present study, the expression of *TRPA1* was higher in the ovaries than in the testes (Figs. 5 and Additional file 5: Figure S2), indicating the involvement of *TRPA1* in the ovarian development of *P. trituberculatus*.

*BAP1* is an important nuclear ubiquitin hydrolase involved in the cell cycle regulation, cell proliferation, cellular differentiation, repair of DNA damage, and apoptosis [40]. Ubiquitin C-terminal hydrolase was found to be involved in sex differentiation in fish [41]. The function of this protein remains undetermined despite numerous studies conducted on ubiquitin hydrolases. Some researchers suggest that ubiquitin hydrolases play an important role in sperm acrosomal function and antipolyspermy defense during porcine fertilization [41]. The qRT-PCR result indicated that the expression of *BAP1* was increased in the stage II, and the expression level was significantly higher in the ovary (*p* < 0.05) than in the testes (Fig. 5 and Additional file 5: Figure S2). This preliminary result showed that *BAP1* may play a crucial role in oogenesis and ovarian development. However, its physiological function needs further investigation.

## Conclusions

A total of 135,992 transcripts and 88,804 unigenes were obtained among which were many genes potentially involved in gonadal development, gametogenesis, and signal transduction. Analysis of DEGs revealed 9066 significant genes between the female and male species in stage II, and 4 DEGs (*ADRA1B*, *BAP1*, *ARL3*, and *TRPA1*) were confirmed by qRT-PCR. This study is the first to report on the transcriptome of the reproductive system during a mating embrace (stage II) in *P. trituberculatu.* This study also provides abundant resources for further research on the molecular basis of reproduction and development in crabs. Future studies would certainly require evaluating the roles of the pathways involved and the gene expression profiles associated with those pathways.

## Methods

### Sample preparation and RNA extraction

A total of 10 pre-pubertal females (distinguished by the differences in the abdominal shape and the coloration of the second-to-last segment of the swimming appendage) [9] and 10 mature male crabs (CW > 100 mm, gonadal maturity) [42] were obtained in September 2006 (close to the mating peak) [43] from a local fishing port in Sheyang in Jiangsu Province. The crabs are transferred to the laboratory in Jiangsu key laboratory for bioresources of saline soils at Yancheng Teachers University and then pairs of crabs (1 male and 1 female) were randomly grouped and reared in ten 200-l aquaria at 25 °C and salinity of 30 for mating observations. When pre-pubertal females (Molt cycle D3-D4) initiated molting the pre-copulatory embrace was formatted by positioning females underneath males (stage II) [10]. Prior to copulation, the paired crabs were placed in an ice bath until anesthetized, and the whole male and female reproductive systems were dissected, frozen in liquid nitrogen and stored at −80 °C. Total RNA was then extracted from the whole male and female reproductive system with TRIzol Reagent (Invitrogen) in accordance with the instructions of the manufacturer and then treated with DNase I. Subsequently, cDNA libraries were generated with the TruseqTM RNA sample prep kit, and index-coded samples were clustered with cBot Truseq PE Cluster Kit v3-cBot-HS. Finally, the male and female libraries were sequenced on Hiseq2000 TruSeq SBS Kit v3-HS.

### Date analysis of de novo sequencing data

To obtain high-quality clean data, raw data were first filtered by removing the reads containing low-quality reads (< 20) and poly-N reads (> 20% reads) in SeqPrep (https://github.com/jstjohn/SeqPrep) and Sickle (https://github.com/najoshi/sickle). De novo transcriptome assembly was accomplished using Trinity (http://trinityrnaseq.sourceforge.net/) [44, 45]. The gene functions of all assembled unigenes were annotated based on the following databases with NR protein sequences, including Swissprot (a manually annotated and reviewed protein sequence database); Protein Information Resource (PIR); Protein Data Bank (PDB). Unigenes annotatation and characteristics of homology search of unigenes against the NR database. Similarly, we obtained GO annotations by Blast2Go (http://www.blast2go.com/b2ghome) [46, 47]. KEGG pathway annotation and KEGG orthology assignments were obtained with the KEGG Automatic Annotation Server [48].

### Analysis of differentially expressed genes

According to the results of all sample and reference genome alignments, the number of reads per kilo-base of exon model per Million (RPKM) mapped reads [49] by RSEM (http://deweylab.biostat.wisc.edu/rsem/) [50] and EdgeR (http://www.bioconductor.org/packages/2.12/bioc/html/edgeR.html) [51] were calculated to obtain the differential expression of genes. By the standard FDR < =0.01, |logFC| > =1, significant differentially expressed genes were selected.

### Quantitative real-time PCR analysis

We chose 4 genes that were differentially expressed in the reproductive system for qRT-PCR verification. In addition, on the basis of the KEGG or GO function, these particular genes were selected to verify whether to conform to the transcriptome data. To compare the gene expression of the immature crabs with that of the mature crabs, we set the pre-pubertal crabs as the control group. The gene-specific primers were designed by Primer Premier 6 (Table 2). PCR reaction was run on the Applied Biosystem 7500 real-time PCR system, using 2 × SYBR Green qPCR Mix as recommended by Aidlab Biotechnologies. The reaction mixtures (25 μL) contained 12.5 μL of 2 × SYBR qPCR Mix, 1 μL of cDNA, 1 μL of forward and reverse primers, and 10.5 μL of RNase-free H_2_O. PCR was performed at 95 °C for 3 min, 40 cycles of 95 °C for 15 s, 60 °C for 15 s, and 72 °C for 25 s. At the end of the reaction, a melting curve was generated. PCR was conducted in 3technological replicates and 2 control groups by using all target genes and β-actin control gene [52]. The data for the 2 samples were calculated as the mean of the relative quality value such that the Cycle Threshold (CT) mean of each gene must be less than 30. Finally, relative gene expression was analyzed using the 2^-ΔΔCT^ method [53, 54].

**Table 2:** Real-time PCR primers used in this study

Gene Name	Forward primer sequence (5′-3′)	Reverse primer sequence (5′-3′)
*β-actin*	ACTGGGACGACATGGAGAAGATC	AAACCTTACCACTCCCGCC
*ADRA1B*	TCTGGTCGCTGTGCGTGAT	ATCAAGACCACCAAGACATCCG
*ARL3*	CATCCCTTGCTCTACATTACTTCC	AAACGACGGGTGCCACAG
*BAP1*	ACCCGCACTCCTCCCTTAT	GAGAACCAAGTGGAGCAGACA
*TRPA1*	AGTAGCGGCAACATGTCCACC	CCAAACTAACCCTGAAAGACCG

## Additional files

Additional file 1: Table S1. Unigenes were mapped to the reference canonical pathways in KEGG. (XLS 1156 kb)

Additional file 2: Table S2. Differentially expressed genes (DEGs) between the ovary and testis. (XLS 1611 kb)

Additional file 3: Table S3. Different up-regulated and down-regulated genes in reproductive system. (XLS 13119 kb)

Additional file 4: Figure S1. qRT-PCR was performed for 4 genes that were identified as differentially expressed between the immature and mature male reproductive systems. The Y axis shows the relative mRNA expression levels. * *p* < 0.05 ** *p* < 0.01. (PDF 1694 kb)

Additional file 5: Figure S2. qRT-PCR was performed for 4 genes that were identified as differentially expressed between the immature and mature female reproductive systems. The Y axis shows the relative mRNA expression levels. * *p* < 0.05 ** *p* < 0.01. (PDF 1820 kb)

## Abbreviations

DEGs: Differentially expressed genes
EN: Endogenous vitellogeic oocyte
EX: Xogenous vitellogenic oocyte
GO: Gene Ontology
KEGG: Kyoto Encyclopedia of Genes and Genomes
MO: Mature oocyte
NCBI: National Center for Biotechnology Information
NO: Nearly-mature oocyte
PDB: Protein data bank
PIR: Protein Information Resource
PR: Previtellogenic oocyte
SNPs: Single nucleotide polymorphisms
SRA: Short Read Archive
SSRs: Simple sequence repeats
