# Supplementary figures and images for: De novo transcriptome sequencing and analysis of male and female swimming crab (Portunus trituberculatus) reproductive systems during mating embrace (stage II)
Source: BMC Genet. 2018 Jan 3;19:3. doi: 10.1186/s12863-017-0592-5 (PMC5753516; doi:10.1186/s12863-017-0592-5)

## MALE

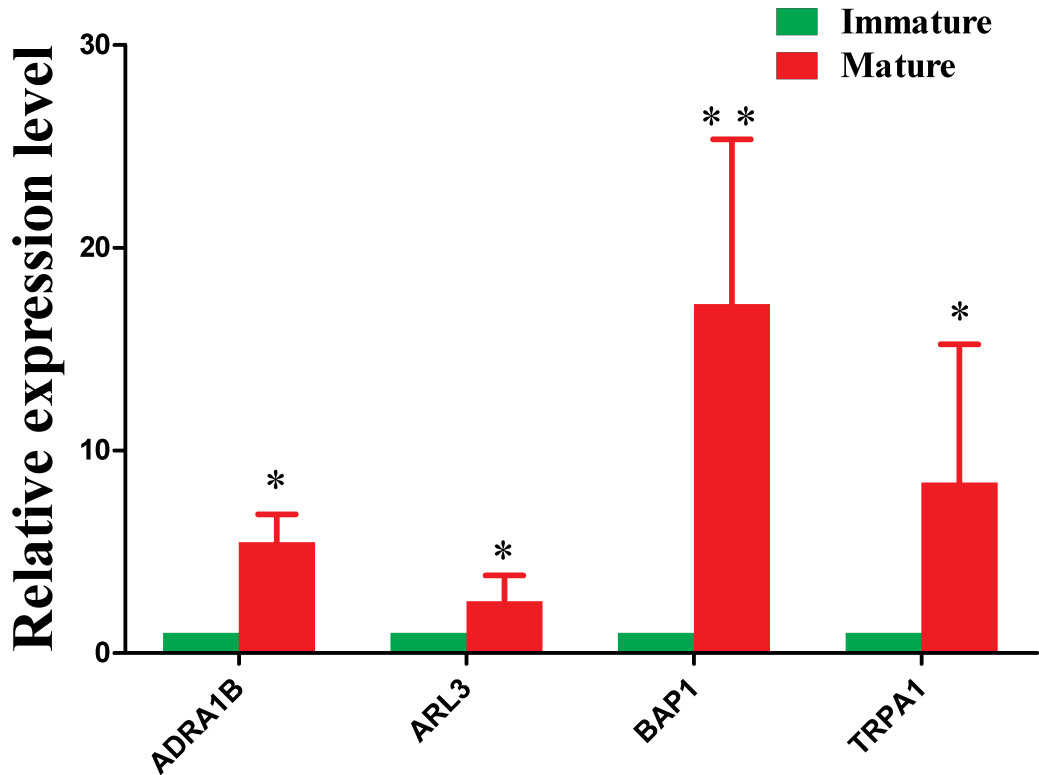

Supplement: Supplementary file 4 — qRT-PCR was performed for 4 genes that were identified as differentially expressed between the immature and mature male reproductive systems. The Y axis shows the relative mRNA expression levels. * p < 0.05 ** p < 0.01. (PDF 1694 kb) [file 12863_2017_592_MOESM4_ESM.pdf]

Relative expression level

FEMALE

Immature  
Mature

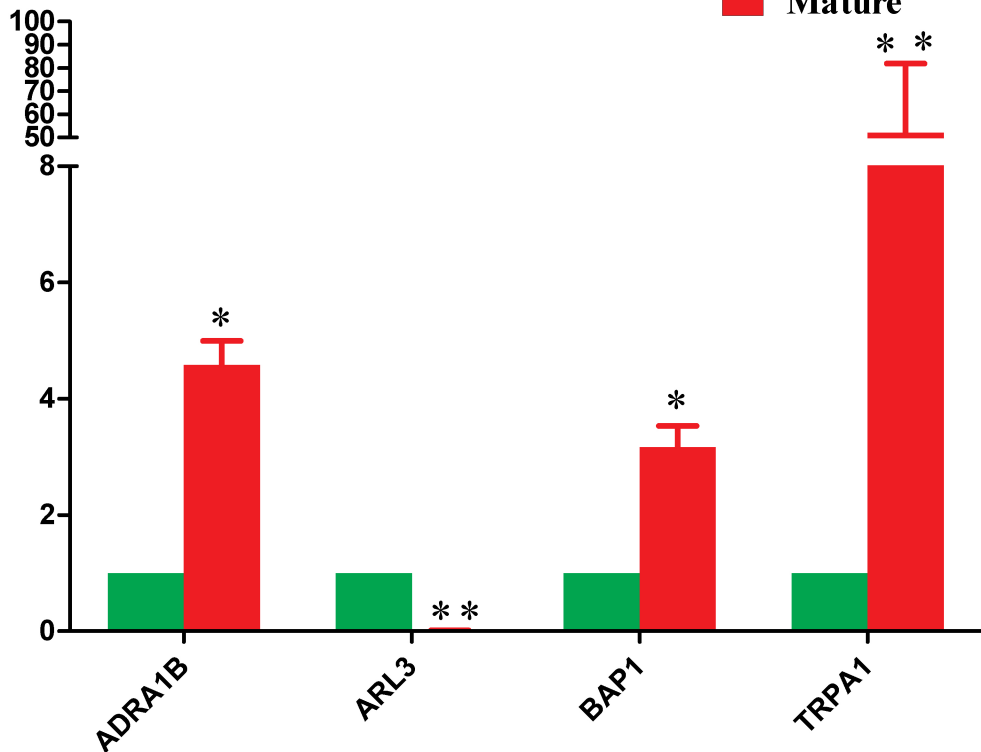

Supplement: Supplementary file 5 — qRT-PCR was performed for 4 genes that were identified as differentially expressed between the immature and mature female reproductive systems. The Y axis shows the relative mRNA expression levels. * p < 0.05 ** p < 0.01. (PDF 1820 kb) [file 12863_2017_592_MOESM5_ESM.pdf]
